# Prevalence of growth retardation among children and adolescents in China: a systematic review and meta-analysis

**DOI:** 10.3389/fped.2025.1634605

**Published:** 2025-12-17

**Authors:** Wei Wang, Zhanpeng Qiu, Fang Wang, Dongsheng Qiu, Guoping Ye

**Affiliations:** 1 Xiamen TCM Hospital Affiliated to Fujian University of Traditional Chinese Medicine, Fujian, Xiamen, China; 2 Fujian University of Traditional Chinese Medicine, Fujian, Fuzhou, China; 3 Waigang Town Community Health Service Center, Jiading District, Shanghai, China; 4 Xiamen Hospital, Dongzhimen Hospital, Beijing University of Chinese Medicine, Fujian, Xiamen, China

**Keywords:** growth delay, stunting, children, adolescents, prevalence, meta-analysis

## Abstract

**Objective:**

The objective of this study was to determine the prevalence of growth delay in Chinese children and adolescents through a meta-analysis, providing scientific evidence for early intervention and prevention.

**Methods:**

Relevant studies on the prevalence of growth delay in Chinese children were retrieved from eight major databases: China National Knowledge Infrastructure (CNKI), Wanfang, Chongqing VIP, Chinese Biomedical Literature Database, PubMed, Embase, Cochrane Library, and Web of Science. Two researchers independently assessed the quality of the studies based on the inclusion criteria for cross-sectional studies outlined in the STROBE guidelines. Any discrepancies were resolved through cross-checking. Data extracted from studies were analyzed using Stata 15.

**Results:**

A total of 50 studies were included, with a sample size of 2,644,818 participants. The meta-analysis revealed that the overall prevalence of growth delay in Chinese children and adolescents was 5.7% (95% CI: 5.3%–6.2%). Subgroup analysis by age demonstrated the following prevalence rates: 7.4% (95% CI: 6.6%–8.3%) for ages 0–2 years, 6.8% (95% CI: 6.1%–7.4%) for ages 3–6 years, 3.9% (95% CI: 3.6%–4.2%) for ages 6–12 years, and 3.0% (95% CI: 2.3%–3.6%) for ages 13–18 years. Statistically significant differences were observed between age groups (*P* < 0.05). With regard to gender, the prevalence was 6.2% (95% CI: 5.4%–7.0%) for males and 6.6% (95% CI: 5.7%–7.5%) for females, with no significant difference between genders (*P* > 0.05). Analysis by residential area indicated that the prevalence in rural areas was 8.4% (95% CI: 6.2%–10.5%), compared to 3.5% (95% CI: 2.5%–4.4%) in urban areas, showing a statistically significant difference (*P* < 0.05). Geographically, the Southwest region had the highest prevalence at 9.2% (95% CI: 4.4%–14%), followed by South China (7.0%, 95% CI: 5.7%–8.3%), Northwest China (5.7%, 95% CI: 2.5%–9.0%), Central China (3.8%, 95% CI: 1.4%–6.1%), East China (2.6%, 95% CI: 2.0%–3.2%), and North China (1.8%, 95% CI: 1.1%–2.4%). No significant differences were found in growth delay prevalence among regions (*P* > 0.05). In terms of study year, the prevalence was 25.8% (95% CI: 2.2%–49.5%) in 2005–2009, 5.2% (95% CI: 3.9%–6.6%) in 2010–2019, and 3.0% (95% CI: 2.7%–3.3%) in 2020–2024, with statistically significant differences observed across years (*P* < 0.05).

**Conclusion:**

The prevalence of growth delay in Chinese children and adolescents is gradually decreasing, with variations across age groups and residential environments. Efforts to alleviate this issue should include enhanced public health awareness, promotion of healthy lifestyles, improved family nutrition education and training, and strengthening of the healthcare system.

**Systematic Review Registration:**

CRD42024579022.

## 1 Introduction

The physical development of children is not only an important indicator of their nutritional and health status but also serves as a key marker for the socioeconomic development of a country or region (1, 2). With ongoing socioeconomic development, the nutritional status of children has significantly improved. However, the issue of growth retardation among children in developing countries remains a public health concern that warrants attention (3). According to a 2020 survey by the World Health Organization (WHO) (4), 149 million children globally are affected by growth delays, with a prevalence rate of 22%. The 2020 survey on the nutritional status of Chinese residents (5) revealed that the prevalence of growth retardation was 4.8% among children under 6 years of age and 2.2% among those aged 6–17 years. In rural areas, the prevalence of growth retardation among children and adolescents is approximately 2–3 times higher than that in urban areas. The causes of growth retardation in children are complex and diverse, often closely related to genetics, endocrine factors, and insufficient nutritional intake (6). Growth retardation directly impacts the physical and mental health of children and adolescents, increasing the risk of chronic diseases such as obesity, cardiovascular disease, and diabetes in adulthood. It also has long-term negative effects on cognitive and socioemotional development and may even lead to depression. In severe cases, it may lead to developmental disorders affecting various organs and tissues, as well as deficiencies in immune and neurological function (7, 8). With recent advancements in diagnostic and therapeutic technologies, together with ongoing economic development, the incidence of growth retardation in Chinese children has demonstrated a downward trend. Furthermore, at the international level, childhood nutritional intervention is considered one of the most valuable investments in human capital (9). In China, national-level censuses are conducted intermittently, while localized and regional studies are conducted annually. These studies ensure that incidence data remain temporally relevant, underscoring the importance of regularly incorporating new evidence to update the prevalence of growth retardation among Chinese children. Such efforts enable a timely and comprehensive understanding of the overall status, trends, and influencing factors related to physical development in this population. Accordingly, this study employs a meta-analytic approach to update the current epidemiological profile of growth retardation in Chinese children and investigate its potential influencing factors in depth, thereby providing an evidence base for the development of more targeted intervention strategies.

## 2 Materials and methods

This report adhered to the Preferred Reporting Items for Systematic Reviews and Meta-Analyses (PRISMA) guidelines (10), and the study protocol was registered and published on PROSPERO [CRD42024579022].

### 2.1 Literature search

A literature search was conducted on the prevalence of growth retardation and stunting in Chinese children in both Chinese and English databases. The Chinese databases included China National Knowledge Infrastructure (CNKI), Wanfang, Chongqing VIP, and China Biology Medicine disc (CBMdisc). The English databases included PubMed, Embase, Cochrane Library, and Web of Science. The search covered the period from the inception of each database to 20 September 2024, without language restrictions but limited to studies conducted in China. In addition, references from systematic reviews and gray literature were also screened to avoid missing relevant studies. The following Chinese search terms were included: “儿童,” “青少年,” “生长迟缓,” “矮小,” “患病率,” “流行率,” “流行病学调查.” For the English search, both subject terms and free terms were used, including “Adolescent,” “Child,” “Growth Disorders,” “Stunting,” “epidemiology,” “Prevalence,” “China,” and “Chinese.” The search strategies were adjusted according to the characteristics of each database, and cross-database searches were conducted to ensure comprehensiveness. For example, the search strategy for PubMed is presented in Table 1, additional search strategies available in Supplementary 1.

**Table 1:** PubMed search strategies for growth retardation prevalence in Chinese children and adolescents.

Steps	Retrieval formula
#1	“Adolescent” [Mesh] OR “Adolescents” [Title/Abstract] OR “Adolescence” [Title/Abstract] OR “Female Adolescent” [Title/Abstract] OR “Male Adolescent” [Title/Abstract] OR “Youth” [Title/Abstract] OR “Teen” [Title/Abstract] OR “Teenager” [Title/Abstract] OR “Child” [Mesh] OR “children” [Title/Abstract]
#2	Growth Disorders[Mesh] OR “Stunted Growth” [Title/Abstract] OR “Growth, Stunted” [Title/Abstract] OR “Growth Disorder” [Title/Abstract] OR “Stunting” [Title/Abstract] OR “Stuntings” [Title/Abstract]
#3	“China” [Mesh] OR “chinese” [Title/Abstract]
#4	“Prevalence” [Mesh] OR “Prevalences” [Title/Abstract] OR “Point Prevalence” [Title/Abstract] OR “Period Prevalence” [Title/Abstract] OR “Period Prevalences” [Title/Abstract] OR “epidemiology” [Subheading] OR “epidemics” [Title/Abstract] OR “incidence” [Title/Abstract] OR “morbidity” [Title/Abstract] OR “occurrence” [Title/Abstract]
#5	#1 AND #2 AND #3 AND #4

### 2.2 Inclusion and exclusion criteria

The inclusion criteria were as follows: (1) studies conducted in China with participants aged ≤18 years; (2) observational or cross-sectional studies with clear sample size and reported prevalence or number of cases; (3) clearly defined diagnostic criteria and methods; and (4) sample size ≥5,000 participants.

The exclusion criteria were as follows: (1) studies with incomplete data or duplicated publications; (2) studies with inadequate design or statistical errors; and (3) reviews, conference abstracts, and similar publications.

### 2.3 Literature screening and data extraction

The identified studies were first input into EndNote 20 software for automatic screening and removal of duplicates. Two independent researchers (WW and FW) then screened the deduplicated articles. The titles and abstracts of the articles were initially reviewed, followed by downloading and full-text review of the studies that met the inclusion criteria after initial screening. During this process, all excluded articles were recorded, with rational explanations provided for exclusion. Any disagreements during this process were resolved through discussion or adjudicated by a third senior researcher. The final screening results were cross-checked by two researchers.

The following information or data were extracted from the included studies: (1) study characteristics: first author, publication year, geographical location (province), region (urban/rural), sample size, and diagnostic criteria; (2) participant characteristics: age, gender, and origin; and (3) outcome measures: prevalence (by gender, region, and age). If any information required for extraction was missing or not clearly reported in the studies included in the systematic review, the researchers attempted to contact the authors to obtain the data or directly retrieved the original studies for supplementation. Any disagreements that arose during the process of data extraction were resolved through discussion or adjudicated by a third senior researcher.

### 2.4 Literature quality assessment

The quality of the included studies was assessed using the STROBE checklist (11) for cross-sectional studies, which consists of 22 items covering the Introduction, Methods, Results, and Discussion sections. Each item that met the criteria was assigned 1 point, and those that did not were assigned 0 points, with a total score of 22 points. A score of ≤5 was considered low quality, a score of 6–11 was considered moderate quality, and a score >11 was considered high quality.

## 3 Results

### 3.1 Basic characteristics of included studies

**Figure 1:**
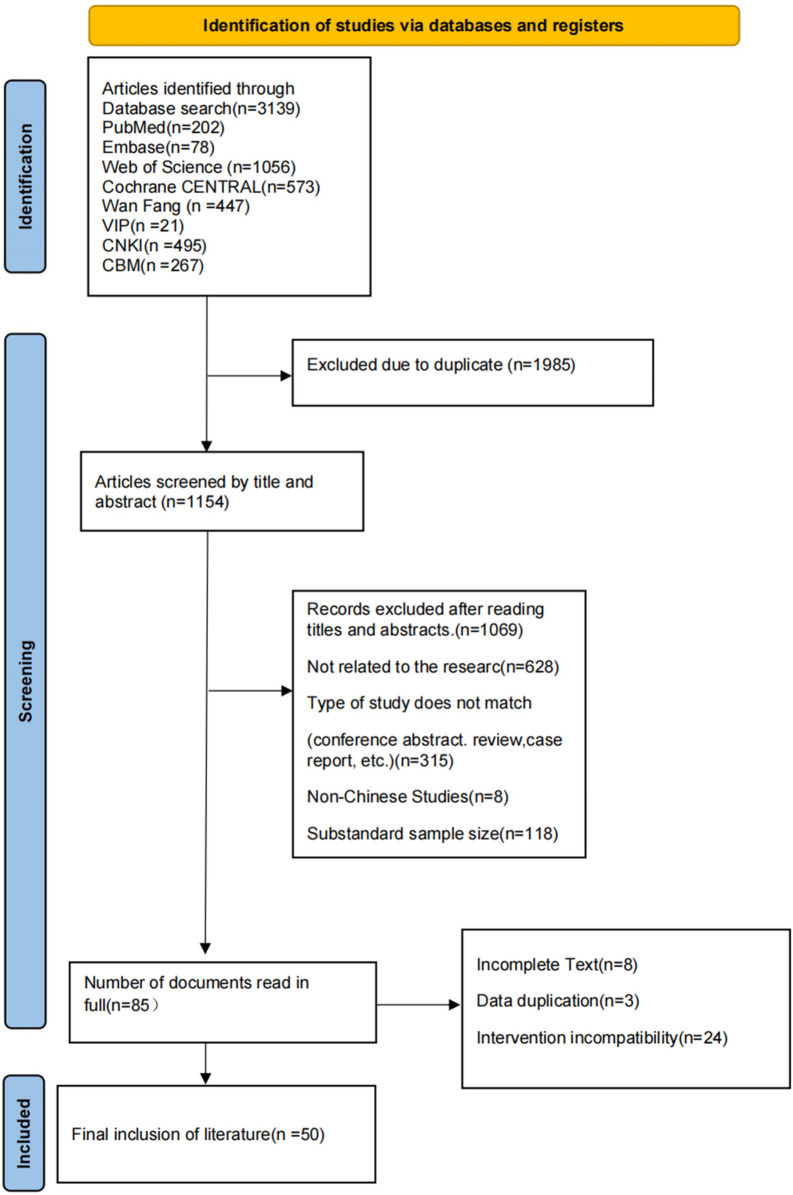
Flowchart of identification and screening for studies on growth retardation prevalence in Chinese children and adolescents.

A total of 3,139 articles were initially retrieved. After screening the titles and abstracts and conducting a full-text review, 50 studies (12–61) were included in the final analysis. Of these, 45 were in Chinese (12–14, 16–22, 24–40, 42–48, 50–53, 55–61) and five were in English (15, 23, 41, 49, 54). The literature screening process and results are shown in Figure 1. The studies covered data from 31 provinces and four direct-controlled municipalities across China. The total sample size across all studies was 2,644,818 participants, among whom 77,586 cases of growth retardation were identified.

### 3.2 Quality assessment of included studies

Two studies (24, 26) scored 5 points and were classified as low quality. Thirty-nine studies (12–22, 25, 27–31, 33–36, 38–40, 42, 44–48, 50, 52, 53, 55–58, 60, 61) scored between 6 and 11 points and were classified as moderate quality. Nine studies (23, 32, 37, 41, 43, 49, 51, 54, 59) scored above 11 points, with the highest score being 15. The average score across the studies was 8.4 points (Table 2).

**Table 2:** General information and quality scores of the included studies.

Author	Year	Investigation period	Area	Regions	Diagnostic criteria	Sampling methods	Age (Year)	Case	Overall	Boys (case/overall)	Girls (case/overall)	Urban (case/overall)	Rural (case/overall)	STROBE Scores
Chen	2005	2000–2004	Jinjiang	South China	4	Random	0–7	9,503	20,493	—	—	—	—	7
Dang	2005	2000–2004	40 counties in Tibet	Southwest Region	2	Stratified random	0–3	1,668	7,252	1,004/4,112	664/3,140	—	—	8
Cheng	2009	2003	Shanghai	East China	2	Cluster sampling	6–18	2,658	70,431	1,172/34,449	1,486/35,982	836/30,121	1,822/40,310	10
Wang	2009	2006	13 provinces in the Midwest	-	2	Stratified random	0–5	2,428	8,041	1,437/4,463	991/3,578	—	—	10
Zhang	2011	2011	Meizhou	South China	2	Cluster sampling	0–5	195	12,928	108/6,769	87/6,159	—	—	6
Liu	2013	2013	Ningbo	East China	4	Stratified cluster random	0–7	581	8,006	333/4,139	248/3,867	104/2,506	477/5,500	11
Wang	2013	2012	Sichuan	Southwest Region	2	Random cluster	0–5	5,634	64,038	2,894/33,422	2,740/30,616	—	—	8
Xiang	2014	2010–2011	Chongqing	Southwest Region	3	Cluster	6–18	16,644	70,952	5,671/36,427	10,973/34,525	7,164/42,789	9,480/28,163	8
Wu	2015	2011	Beijing	North China	2	Random cluster	6–19	2,630	99,482	1,425/50,889	1,205/48,593	—	—	12
Li	2015	2013	Yan'an	Northwest China	2	Cluster	7–13	770	8,043	339/4,043	431/4,000	370/4,652	400/3,391	6
Chen	2016	2014	Dongguan	South China	2	Cluster	3–6	748	18,484	—	—	—	—	6
Jing	2016	2012–2014	Chongqing	Southwest Region	2	Cluster	3–6	164	17,329	76/8,798	88/8,531	—	—	7
Chen	2017	2014	Anhui	East China	5	Stratified cluster	7–12	172	6,082	85/3,104	87/2,978	68/3,049	104/3,033	6
Liu	2017	2017	Jining	East China	3	Random cluster	6–16	287	9,095	166/4,937	121/4,158	120/4,448	167/4,647	5
Tao	2017	2017	Yizheng	East China	2	Cluster	6–14	210	9,338	—	—	84/6,493	126/2,845	8
Yu	2017	2015	Wuhu	East China	2	Cluster	3–6	449	89,060	—	—	176/43,649	273/45,411	6
Qin	2017	2016	Wuhan	Central China	6	Random	3–14	1,640	30,000	789/15,724	851/14,276	682/16,992	958/13,008	10
Xu	2017	2017	Jingdezhen	Central China	2	Cluster	6–12	194	10,436	91/5,267	103/5,169	118/6,733	76/3,703	5
Fang	2018	2010–2012	31 inland provinces	-	1	Cluster random	6–17	1,154	36,058	654/18,204	500/17,854	—	—	7
Sang	2018	2018	Datong	North China	5	Random cluster	7–18	272	14,179	128/6,858	144/7,321	—	—	12
Wang	2018	2015	Taicang	East China	2	Cluster	3–14	1,174	63,049	640/34,171	534/28,878	185/20,192	989/42,857	11
Shi	2019	2017–2019	Shaanxi	Northwest China	5	Cluster	3–6	279	8,719	118/4,358	161/4,361	—	—	6
Zhu	2019	2018	Yunnan	Southwest Region	2	Stratified cluster	0–5	139	5,394	—	—	87/2,358	52/3,036	8
Zhang	2020	2016	9 cities in China	-	3	Stratified cluster sampling	<7	2,141	110,499	1,121/57,921	1,020/52,578	904/55,524	1,237/54,975	13
Zhang	2020	2017	Southern Tianjin	North China	3	Cohort study	3.5–6.5	75	6,757	40/3,624	35/3,133	—	—	6
Hong	2020	2018	Suzhou	East China	5	Cluster sampling method	6–12	2,385	82,508	1,168/44,779	1,217/37,729	731/39,745	1,654/42,763	12
Lin	2020	2019	Xiamen	South China	2	Random cluster	1–6	391	43,586	—	—	—	—	6
Xiong	2020	2018	Rong'an	South China	4	Random cluster sampling	6–12	700	8,729	395/4,452	305/4,277	—	—	7
Guo	2020	2018–2019	Baota District, Yan'an	Northwest China	2	Cluster sampling	7–12	525	7,300	245/3,906	280/3,394	—	—	7
Gou	2020	2016–2018	Chengdu	Southwest Region	2	Cluster	1–7	1,115	99,190	645/51,583	470/47,607	—	—	6
Liu	2021	2021	7 provinces in China	-	2	Random cluster sampling	<2	675	17,193	446/8,927	229/8,266	—	—	12
Zhu	2021	2018	5 cities in Shanxi	North China	2	Stratified cluster random sampling	<5	87	6,407	41/3,227	46/3,180	—	—	8
Zhao	2021	2018–2020	Jiading	East China	4	Cluster	0–3	11	8,262	—	—	—	—	6
Huang	2021	2020	Pingshan	South China	5	Whole random	6–14	538	12,504	286/6,906	252/5,598	—	—	7
Yin	2021	2020	Foshan	South China	2	Whole	3–6	39	32,165	—	—	—	—	6
Wang	2021	2019	Xinjiang Production	Northwest China	2	Cluster	<7	585	9,051	—	—	—	—	6
Zheng	2021	2014–2018	Lanzhou	Northwest China	2	Whole	0–5	4,602	671,394	—	—	—	—	6
Luo	2021	2019	Sichuan	Southwest Region	2	Whole random	<5	384	7,534	229/3,901	155/3,633	71/3,025	313/4,509	14
Yu	2022	2020.03	Weifang	East China	4	Cluster sampling	4–15	166	8,740	74/4,675	92/4,065	—	—	7
Zhang	2,022	2018–2021	Suzhou	East China	2	Cluster	1–5	3,183	319,791	1,071/154,253	2,112/165,538	—	—	8
Wang	2022	2020–2022	Hainan	South China	2	Stratified random sampling	0–5	256	8,244	145/4,282	111/3,962	50/2,266	206/5,978	15
Liu	2022	2017–2019	10 cities in Henan Province	Central China	5	Cluster sampling	3–18	662	12,667	361/6,569	301/6,098	—	—	9
Li	2,022	2019.08–11	Rural Hunan	Central China	2	Stratified cluster sampling	<6	241	5,529	104/2,806	137/2723	—	—	13
Zhang	2022	2019–2020	Hanzhong	Northwest China	5	Whole	3–12	857	11,690	431/6,153	426/5,537	412/6,915	445/4,775	13
Shi	2023	2021	Dongying	East China	2	Whole	6–12	1,541	85,376	769/44,850	772/40,526	851/45,734	690/39,642	7
Wu	2023	2018–2020	Xuzhou	East China	2	Cluster	2–6	350	6,820	170/3,593	180/3,227	—	—	6
Pan (1)	2023	2018–2022	Shaoguan	South China	2	Cluster	0–3	656	17,542	—	—	—	—	9
Pan (2)	2023	2020–2022	Shaoguan	South China	2	Whole sampling	3–6	861	18,139	465/9,614	396/8,525	—	—	9
Zhang	2023	2019–2020	Panyu	South China	2	Whole	0–6	4,928	432,085	—	—	—	—	11
Zhu	2023	2019–2021	Zhengzhou	Central China	2	Random cluster	3–6	39	6,938	18/3,562	21/3,376	—	—	8

Diagnostic Criteria: 1, height below the age-specific mean by 1 SD; 2, height below the age-specific mean by 2 SD; 3, growth delay: height below the age-specific mean by 2 SD; Short stature: Height below the age-specific mean by 3 SD; 4, mild: height below the age-specific mean by 1 SD; moderate: <2 SD; Severe: <3 SD; 5, height below the age-specific mean by 3 SD; 6, combined results from imaging, blood tests, and height measurements.

### 3.3 Meta-analysis

#### 3.3.1 Prevalence of growth retardation

**Figure 2:**
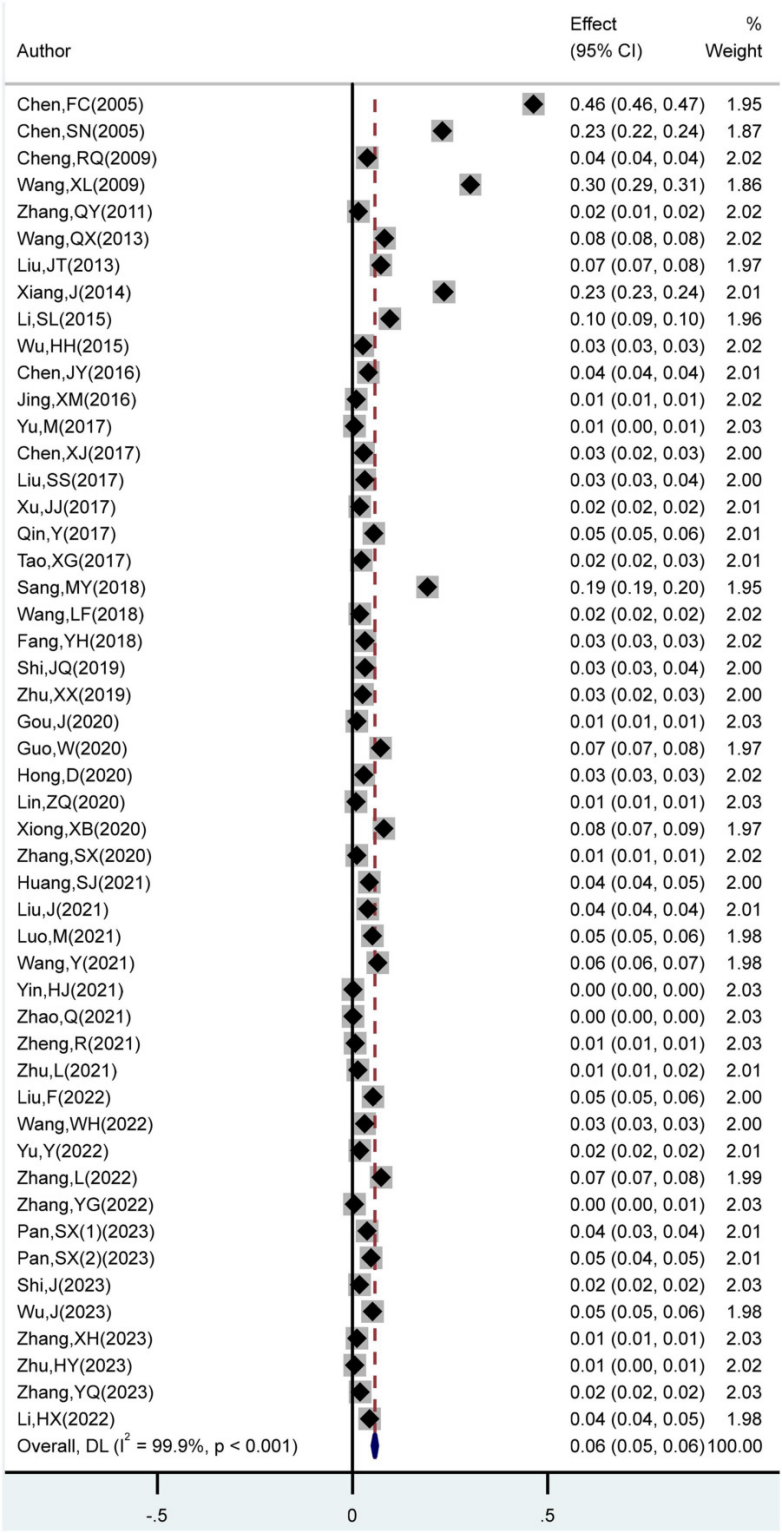
Forest plot of meta-analysis of the total prevalence of growth retardation in Chinese.

A total of 50 studies were included in this analysis, all of which reported the prevalence of growth retardation. The total sample size across the studies was 2,644,818 children and adolescents aged 0–18 years, with 77,586 cases of growth retardation identified. Heterogeneity testing across the 50 studies revealed an *I*^2^ of 99.9% (*P* < 0.05), indicating high heterogeneity. A random-effects model was applied for the meta-analysis, which showed that the overall prevalence of growth retardation among children and adolescents was 6.0% (95% CI: 5.0%–6.0%) (Figure 2).

#### 3.3.2 Subgroup analysis

Owing to considerable heterogeneity among studies, subgroup analysis was performed based on gender, age, residential environment (urban/rural), region, and year of publication. After testing, it was found that heterogeneity remained significant; therefore, a random-effects model was used for pooled analysis (Tables 3, 4).

1. A total of 35 studies involving children and adolescents of different ages were included in the analysis of growth retardation. The highest prevalence was observed in infants aged 1 year, at 8.9% (95% CI: 7.3%–10.5%), while the lowest prevalence was found in adolescents aged 15 and 16 years, at 1.9% (95% CI: 1.0%–2.9%) and 1.9% (95% CI: 1.1%–2.7%), respectively. The differences in growth retardation prevalence across different age groups were statistically significant (*P* < 0.05) (Table 3).

**Table 3:** Subgroup analysis of growth retardation prevalence: results by age.

Age	No. of articles	No. of participants	No. of cases	Prevalence (95% CI)	*I*^2^%	*P-*value
0–	11	80,374	3,098	4.6% (3.5%–5.8%)	98.7	
1–	15	93,393	4,510	8.9% (7.3%–10.5%)	99.7	
2–	15	75,438	3,661	8.7% (7.1%–10.3%)	99.7	
3–	18	87,500	3,897	8.0% (6.5%–9.4%)	99.6	
4–	18	115,613	4,509	7.8% (6.4%–9.3%)	99.6	
5–	12	118,799	3,516	6.9% (5.4%–8.3%)	93.4	
6–	18	73,822	2,900	6.4% (5.0%–7.9%)	96.3	
7–	15	46,669	1,327	3.4% (2.8%–4.0%)	99.8	
8–	16	46,802	1,388	3.7% (3.0%–4.5%)	99.9	
9–	16	47,083	1,430	3.8% (3.0%–4.6%)	96.9	
10–	15	44,720	1,414	3.9% (3.1%–4.7%)	96.5	
11–	15	36,082	1,284	4.4% (3.3%–5.4%)	97.5	
12–	14	29,763	1,105	4.6% (3.5%–5.7%)	96.7	
13–	7	21,990	861	3.5% (1.9%–5.0%)	97.8	
14–	7	17,687	589	3.5% (2.4%–4.6%)	92.2	
15–	5	13,318	270	1.9% (1.0%–2.9%)	90.2	
16–	3	7,902	147	1.9% (1.1%–2.7%)	77.4	
17–	3	7,061	142	2.8% (1%–5.7%)	97.6	
18–	1	2,373	58	2.4% (1.8%–3.1%)	0.0	
Overall	35	966,389	36,106	5.4% (5.1%–5.8%)	98.8	<0.01

**Table 4:** Subgroup analysis of growth retardation prevalence: results by age group.

Age	No. of articles	No. of participants	No. of cases	Prevalence (95% CI)	*I*^2^%	*P-*value
0–2	16	249,205	11,269	7.4% (6.6%–8.3%)	99.6	
3–6	28	395,734	14,822	6.8% (6.1%–7.4%)	99.6	
7–12	21	251,119	7,948	3.9% (3.6%–4.2%)	99.7	
13–18	7	70,331	2,067	3.0% (2.3%–3.6%)	96.4	
Overall	35	966,389	36,106	5.4% (5.1%–5.8%)	98.8	<0.01

Subgroup analysis based on the age groups 0–2, 3–6, 7–12, and 13–18 years showed that the prevalence of growth retardation differed significantly across these age groups (*P* < 0.05) (Table 4).

2. A total of 38 studies on growth retardation in adolescents of different genders were included. The prevalence was 6.2% (95% CI: 5.4%–7.0%) in boys and 6.6% (95% CI: 5.7%–7.5%) in girls. There was no statistically significant difference in the prevalence of growth retardation between genders (*P* > 0.05) (Table 5).
3. A total of 18 studies on growth retardation in adolescents from different residential environments were included. The prevalence was higher in rural adolescents, at 8.4% (95% CI: 6.2%–10.5%), compared to 3.5% (95% CI: 2.5%–4.4%) in urban adolescents. The difference in prevalence between rural and urban environments was statistically significant (*P* < 0.01) (Table 5).
4. A total of 46 studies on growth retardation in adolescents from different regions were included. The highest prevalence was observed in the Southwest region, at 9.2% (95% CI: 4.4%–14%), followed by South China at 7.0% (95% CI: 5.7%–8.3%), the Northwest region at 5.7% (95% CI: 2.5%–9.0%), Central China at 3.8% (95% CI: 1.4%–6.1%), East China at 2.6% (95% CI: 2.0%–3.2%), and North China at 1.8% (95% CI: 1.1%–2.4%). There was no statistically significant difference in the prevalence of growth retardation between regions (*P* > 0.05) (Table 5).
5. Subgroup analysis by year of survey showed that the prevalence of growth retardation in children and adolescents was 25.8% (95% CI: 2.2%–49.5%) during 2005–2009, 5.2% (95% CI: 3.9%–6.6%) during 2010–2019, and 3% (95% CI: 2.7%–3.3%) during 2020–2024. The differences in the prevalence across different publication years were statistically significant (*P* < 0.01) (Table 5).

**Table 5:** Subgroup analysis of growth retardation prevalence in Chinese children and adolescents.

Variable	Sections	No. of articles	No. of participants	No. of cases	Prevalence (95% CI)	*I*^2^%	*P-*value
Gender	Overall	38	1,383,675	55,325	6.4% (5.8%–7.5%)	99.8	0.86
	Boys	38	709,717	25,354	6.2% (5.4%–7.0%)	99.8	
	Girls	38	673,958	29,971	6.6% (5.7%–7.5%)	99.9	
Setting	Overall	18	685,737	32,482	5.9% (4.8%–6.9%)	99.9	0.10
	Urban	18	337,191	13,013	3.5% (2.5%–4.4%)	99.8	
	Rural	18	348,546	19,469	8.4% (6.2%–10.5%)	99.9	
Region	Overall	46	2,473,027	71,188	5.1% (4.6%–5.5%)	99.9	0.45
	North China	4	126,825	3,064	1.8% (1.1%–2.4%)	98.6	
	East China	13	740,119	13,167	2.6% (2.0%–3.2%)	99.8	
	South China	11	604,406	18,815	7.0% (5.7%–8.3%)	100.0	
	Central China	5	55,134	2,776	3.8% (1.4%–6.1%)	99.7	
	Northwest China	6	699,435	7,618	5.7% (2.5%–9.0%)	99.8	
	Southwest Region	7	247,108	25,748	9.2% (4.4%–14%)	100.0	
Year	Overall	50	2,644,818	77,586	5.7% (5.3%–6.2%)	99.9	<0.01
	2005–2009	4	78,472	16,257	25.8% (2.2%–49.5%)	100.0	
	2010–2019	19	509,706	33,336	5.2% (3.9%–6.6%)	99.9	
	2020–2024	27	2,056,640	27,993	3.0% (2.7%–3.3%)	99.8	

### 3.4 Sensitivity analysis

The sensitivity analysis indicated that five studies had a significant impact on the pooled results. These five studies were considered the source of heterogeneity in the prevalence of growth retardation in children and adolescents (Supplementary 2).

### 3.5 Publication bias

**Figure 3:**
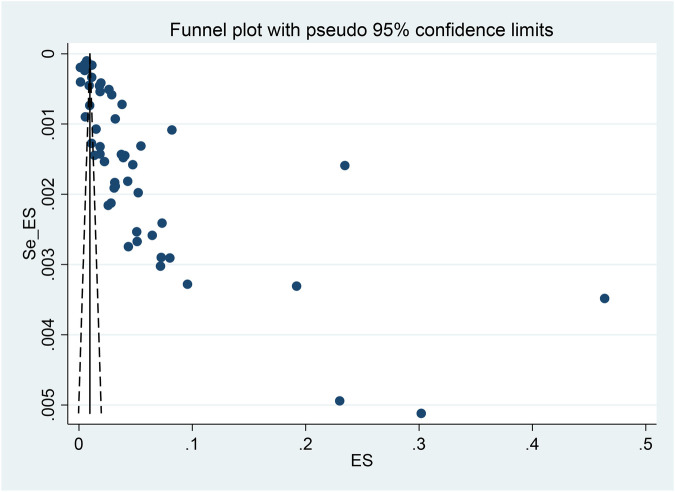
Funnel plot of the enrolled studies.

Funnel plots and Egger's test revealed the presence of publication bias in the studies on the prevalence of growth retardation in children and adolescents (*P* < 0.001). The Egger’s test yielded a result of *t* = 6.14, *P* = 0.017. In addition, the asymmetry of the funnel plot suggested the existence of publication bias in this body of research on the prevalence of growth retardation in children and adolescents (Supplementary 2 and Figure 3).

## 4 Discussion

This study aggregated 50 published research articles on childhood stunting from 2005 to 2024, covering 31 provinces and four municipalities across China, with a total sample size of 2,644,818 children and adolescents. The average AHRQ score of the included studies was 8.4, indicating medium to high quality. The meta-analysis results show that the overall prevalence of growth retardation in Chinese children and adolescents is 5.7% (95% CI: 5.3%–6.2%), which is significantly lower than the prevalence rates in underdeveloped regions like Africa [e.g., Ethiopia 36.6% (62), Rwanda 33% (63)] and Oceania (27%) (3). Previous studies (64, 65) have indicated that countries with higher rates of stunting are typically located near the equator or in the Southern Hemisphere, such as parts of Africa, the Indian subcontinent, and areas between North and South America. In contrast, socioeconomically developed regions like Europe and the U.S. have relatively lower rates of growth retardation (66, 67), largely due to high education levels, well-developed prenatal care, and adequate nutrition, which prevent adverse events during pregnancy and reduce the birth rate of stunted children (68, 69).

In this study, children and adolescents were grouped into age categories (0–2, 3–6, 7–12, 12–18) for subgroup analysis. The prevalence of growth retardation showed a fluctuating trend with age. The lowest rate was found in the 13–18 age group at 3.0%, while the highest was in the 0–2 age group at 7.4%. This could be attributed to the incomplete development of the gastrointestinal function in infants and young children (70), making them more prone to picky eating, indigestion, and bacterial or viral infections (67). Moreover, improper feeding by parents or a lack of awareness regarding height issues in this age group may have contributed to a higher prevalence of stunting. Moreover, maternal health conditions such as malnutrition, diseases during pregnancy, and prematurity are significant factors contributing to growth retardation (71–73).

Subgroup analysis also revealed that the prevalence of stunting in Chinese girls was 6.6%, slightly higher than in boys (6.2%), though the difference was not statistically significant. A study in Iran (74) also suggested no significant difference in stunting rates between boys and girls, and some Asian studies (75) found no significant gender difference either. However, some Chinese studies (18, 21) reported a higher prevalence of stunting in girls, which might be related to the increase in estrogen levels during puberty. Estrogen accelerates bone maturation, which may cause premature closure of growth plates and affect growth speed. In addition, cultural factors, such as the preference for boys over girls in some areas, may contribute to this difference by increasing attention to boys' health.

We also found that the prevalence of growth retardation was significantly higher in rural children (8.4%) compared to urban children (3.5%). This disparity may be explained by advantages in environment, resources, education, and socioeconomic conditions in urban areas (76). Families in some rural areas may face economic constraints, leading to insufficient food supply and difficulty in providing adequate high-quality proteins, vitamins, and minerals to meet children's growth needs, thereby affecting their development. In particular, children in poor regions are more likely to experience malnutrition or micronutrient deficiencies. Furthermore, rural areas often have limited healthcare access, and parents may not pay enough attention to children's health issues, causing delayed diagnosis and treatment for growth-related problems.

The study also demonstrated a significant decline in the prevalence of growth retardation over time in China, decreasing from 25.8% in 2005–2009 to 5.8% in 2010–2019, and further to 3.0% in 2020–2024. This trend is likely attributable to rapid economic development, improvements in nutrition and healthcare, public health policies, and better family education. Collectively, these factors have contributed to better living standards, increased attention to children's nutrition, and a deeper understanding of the adverse effects of growth retardation, thereby improving the overall situation.

In addition, the prevalence of stunting was highest in the Southwest region (9.2%) compared to other regions, with the order of prevalence being Southwest China (9.2%), South China (7.0%), Northwest China (5.7%), Central China (3.8%), West China (2.6%), and North China (1.8%). However, the differences were not statistically significant, likely due to variations in sample sizes and the number of included studies. China's vast geography, diverse ethnicities, economic disparities, environmental conditions, education levels, and cultural practices contribute to regional differences in stunting, necessitating further research.

## 5 Significance and limitations

This study provides significant insights by aggregating data from 31 provinces and four municipalities across China, revealing the trends in the prevalence of growth retardation. It fills the gap of recent data on stunting rates among Chinese children. Despite being a large-sample cross-sectional study, there are some limitations: (1) There was considerable heterogeneity among studies included in this analysis. Although subgroup analysis was performed, the heterogeneity did not decrease significantly. Publication bias was detected through Egger's test and funnel plots, which may affect the accuracy of the meta-analysis results. (2) In terms of diagnostic variability, growth retardation diagnoses in the included studies often relied on measurement indicators, with no uniform laboratory diagnostic standards, which may have contributed to the heterogeneity. (3) With regard to regional representation, no research was available from Northeast China, which limits the representativeness of the analysis. In addition, the impact of ethnic factors on the prevalence of growth retardation was not considered, which may have influenced the results.

## 6 Conclusion

In conclusion, the overall prevalence of growth retardation among Chinese children and adolescents was 5.7%, with significant differences observed across age groups, living environments, and study years. This phenomenon may be attributed to factors such as economic development, education levels, nutrition status, and healthcare conditions. Public health initiatives to promote healthy lifestyles, strengthen family nutrition education, and improve healthcare systems are therefore crucial. This study provides new guidance and data for understanding childhood stunting, offering a reference for policymaking and implementation.

## Data Availability

The original contributions presented in the study are included in the article/Supplementary Material, further inquiries can be directed to the corresponding author.

## Supplementary material

Supplementary 1 S1 File: Search Query.

Supplementary 2 S2 File: Research Indicators and Their Subgroup Plots.
