# Supplementary material for: Prevalence of growth retardation among children and adolescents in China: a systematic review and meta-analysis
Source: Front Pediatr. 2025 Dec 17;13:1634605. doi: 10.3389/fped.2025.1634605 (PMC12754002; doi:10.3389/fped.2025.1634605)
Supplement: Supplementary 1 — S1 File: Search Query. [file Datasheet1.pdf]

1.Age Group：

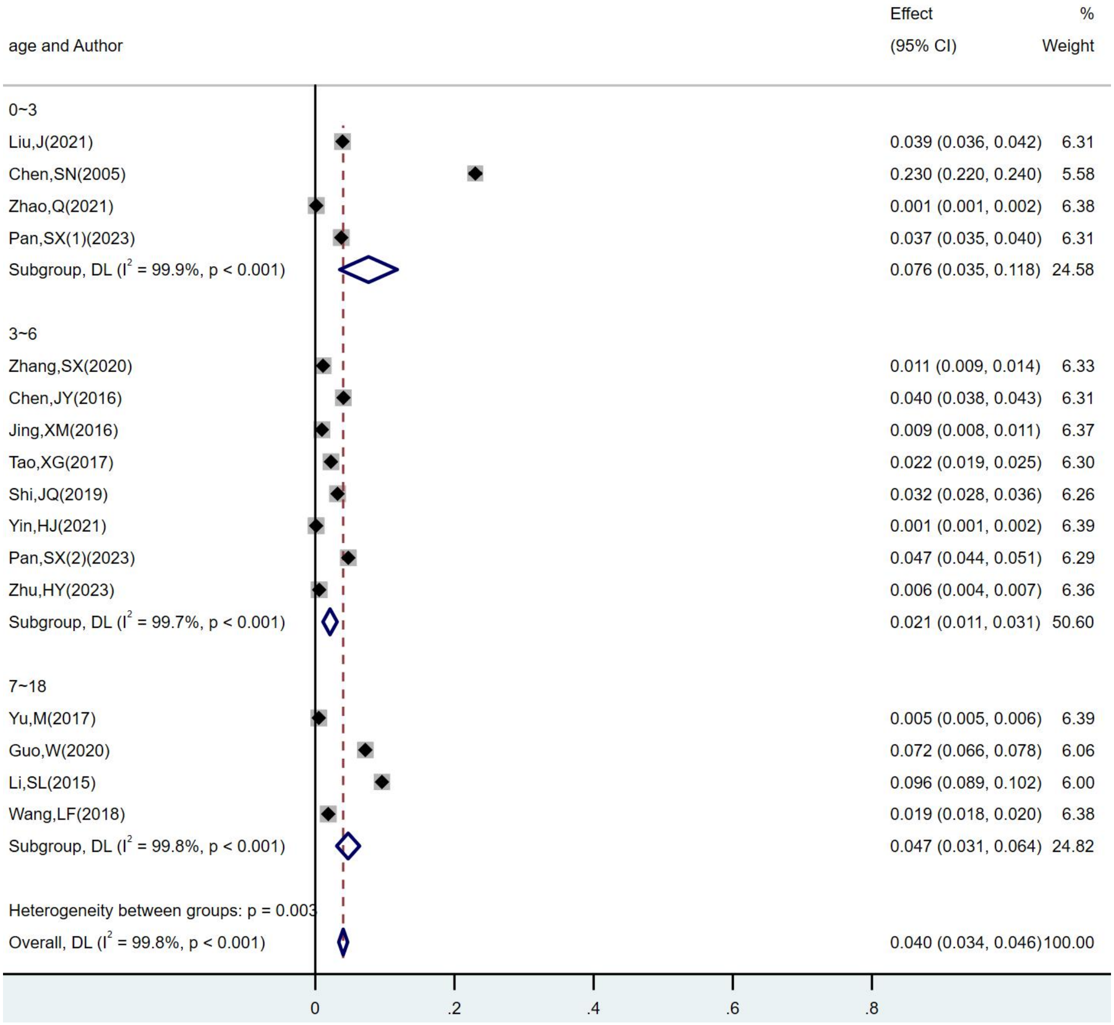

Forest Plot of meta-analysis of the Prevalence of Stunting in Age Groups in China

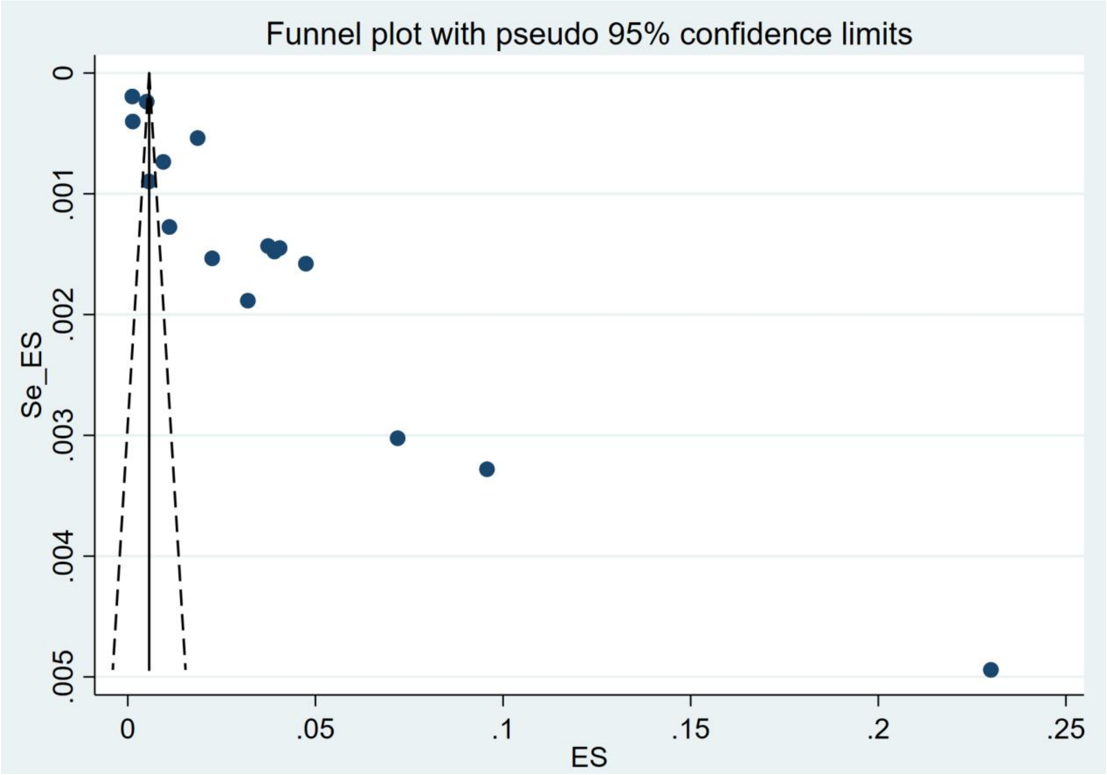

Funnel plot

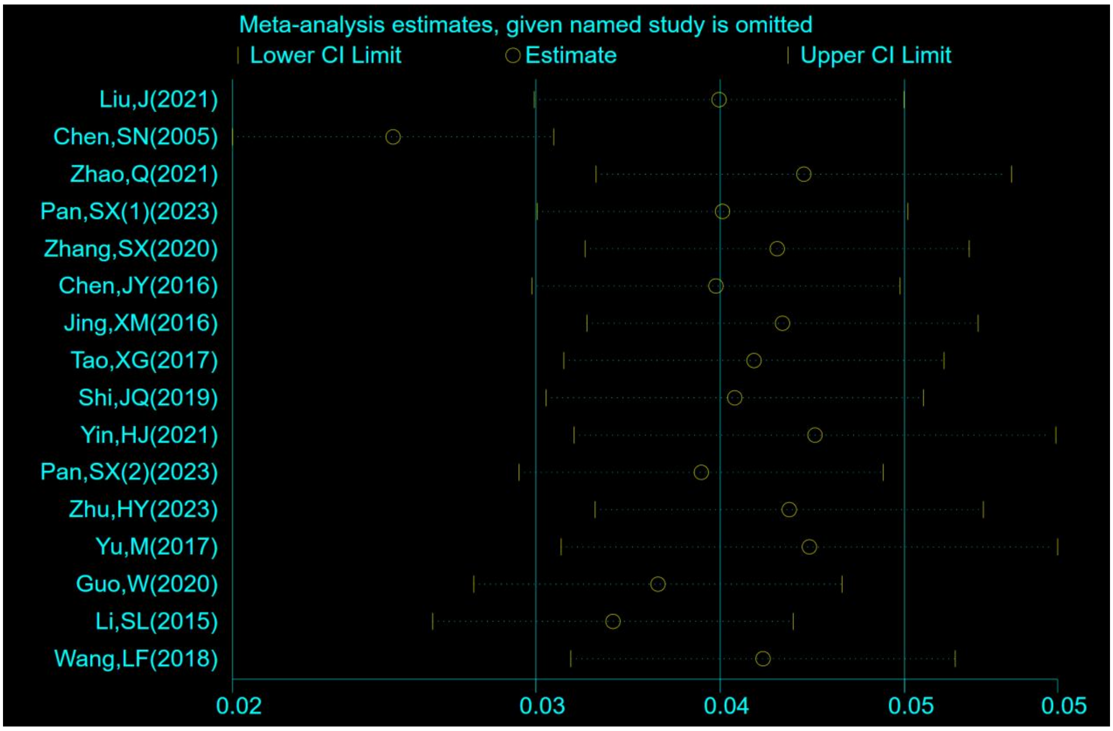

Sensitivity analysis

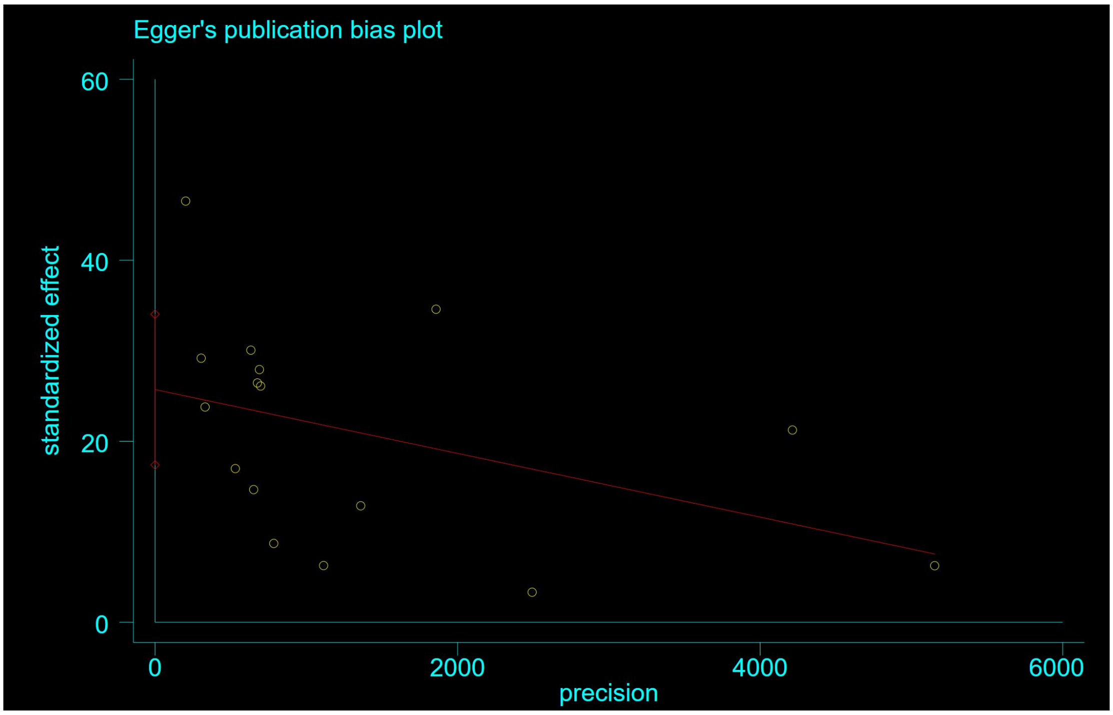

Egger's test1.

```
leta-regression                                      Number of obs   =        16
REML estimate of between-study variance              tau2            =   .003032
; residual variation due to heterogeneity            I-squared_res   =    99.81%
'roportion of between-study variance explained       Adj R-squared   =     4.73%
Joint test for all covariates                        Model F(2,13)   =      1.38
Vith Knapp-Hartung modification                      Prob > F        =    0.2869
```

p	Coef.	Std. Err.	t	P>|t|	[95% Conf.	Interval]
age_1	.0288936	.0390472	0.74	0.472	-.0554628	.1132499
age_2	-.0265463	.0338037	-0.79	0.446	-.0995748	.0464822
_cons	.0477691	.0276057	1.73	0.107	-.0118694	.1074077

Egger's test2.

2.Setting:

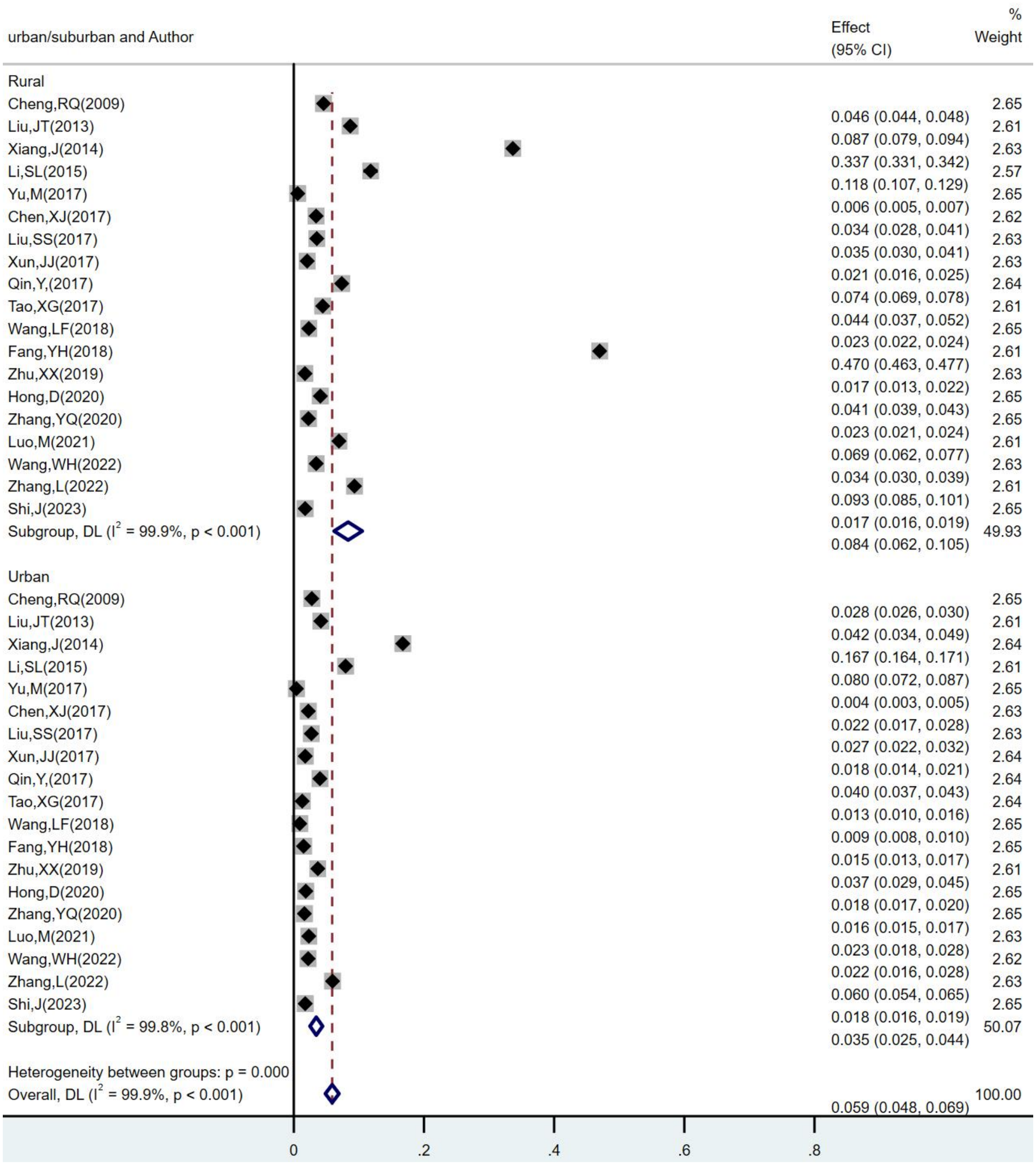

Forest Plot of meta-analysis of the Prevalence of Stunting in setting in China

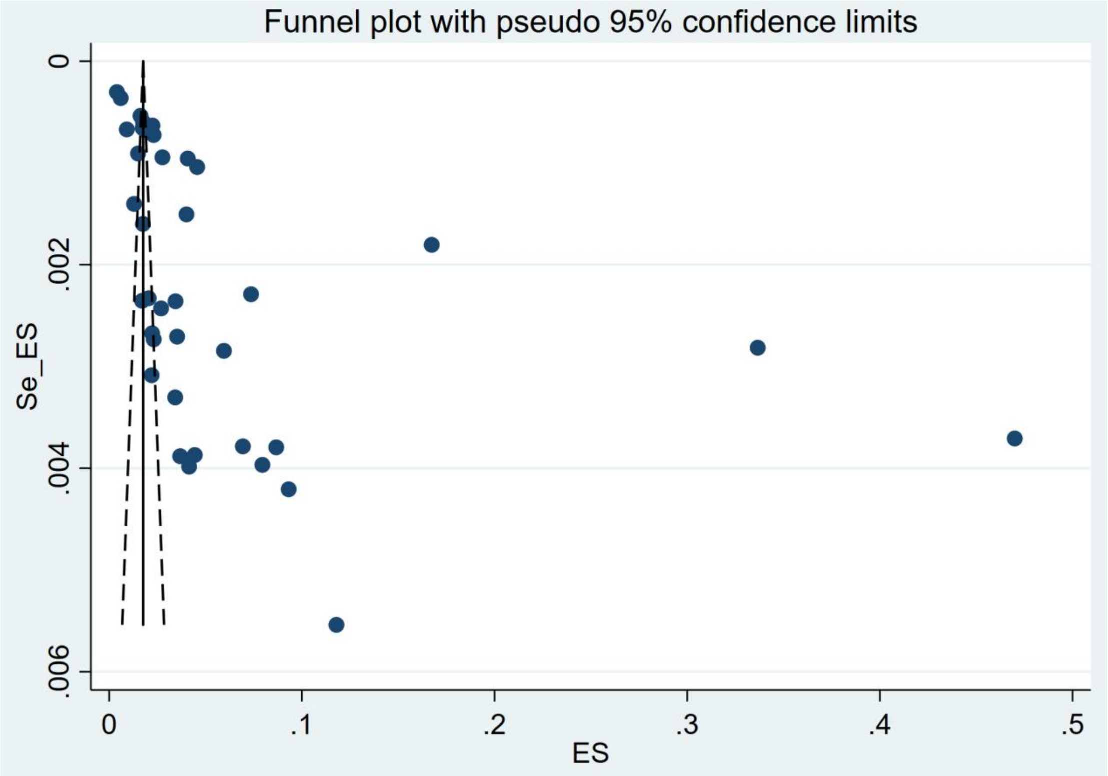

Funnel plot.

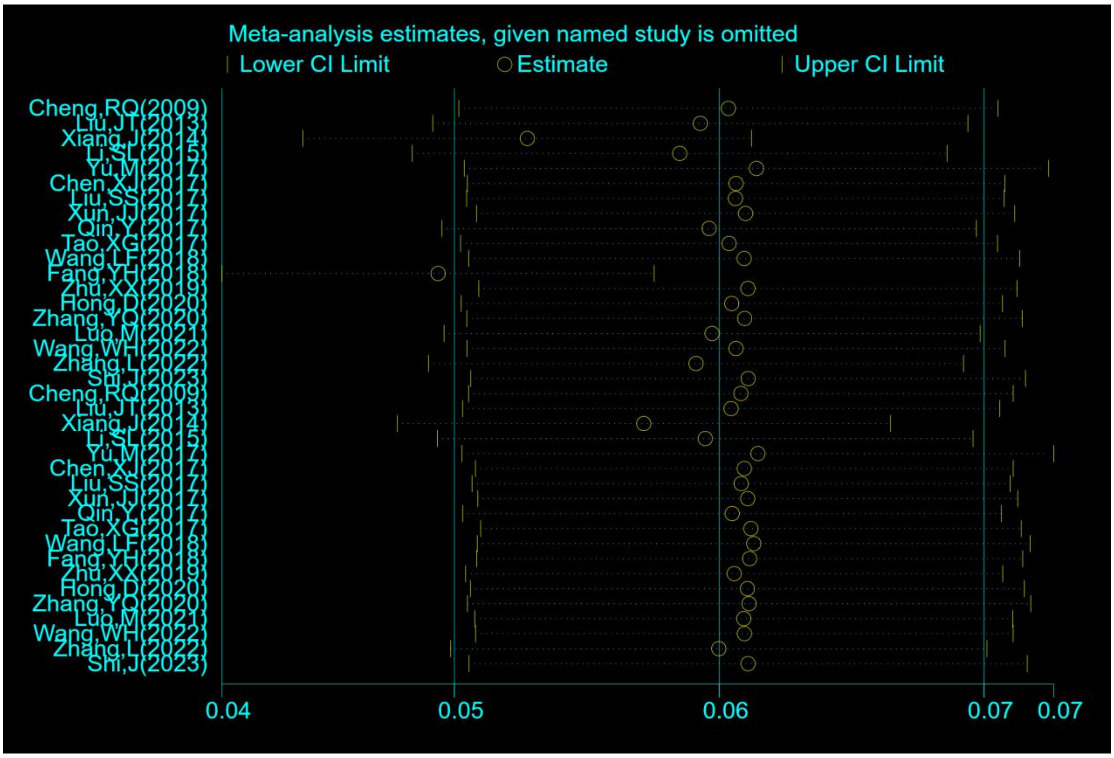

Sensitivity analysis

```
. metareg p (urbansuburban_*) , wsse(se)
note: urbansuburban_2 dropped because of collinearity

Meta-regression                                   Number of obs  =        39
REML estimate of between-study variance           tau2           =    .00753
% residual variation due to heterogeneity         I-squared_res  =    99.91%
Proportion of between-study variance explained    Adj R-squared  =     4.65%
With Knapp-Hartung modification
```

p	Coef.	Std. Err.	t	P>|t|	[95% Conf.	Interval]
urbansuburban_1	.046971	.0278191	1.69	0.000	-.0093959	.1033379
_cons	.0346303	.0199197	1.74	0.000	-.0057309	.0749914

.

Egger's test1

3.Gender:

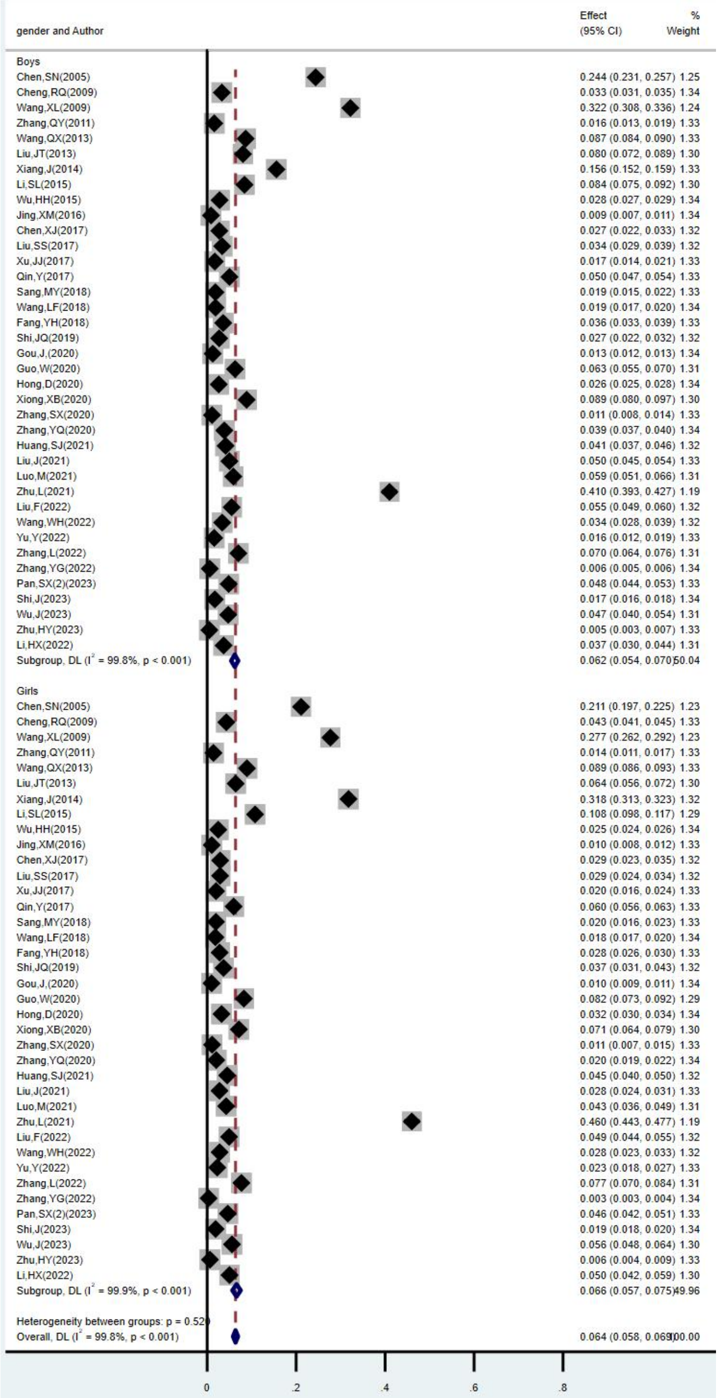

Forest Plot of meta-analysis of the Prevalence of Stunting in Gender in China

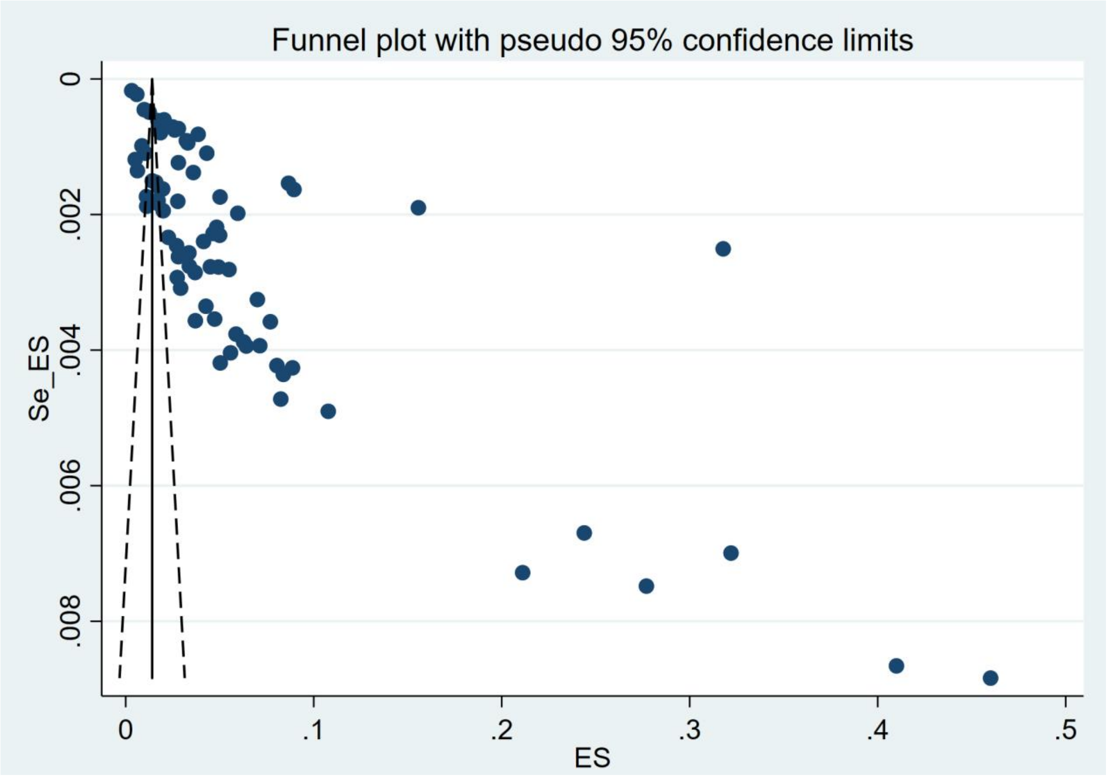

Funnel plot.

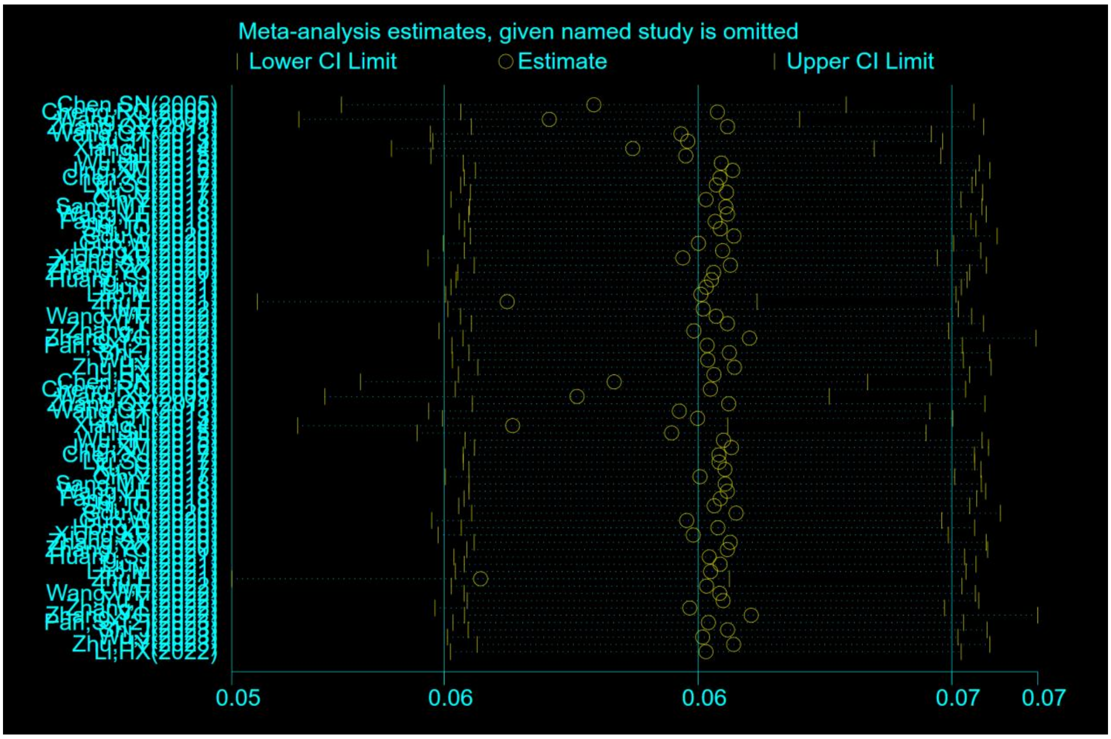

Sensitivity analysis

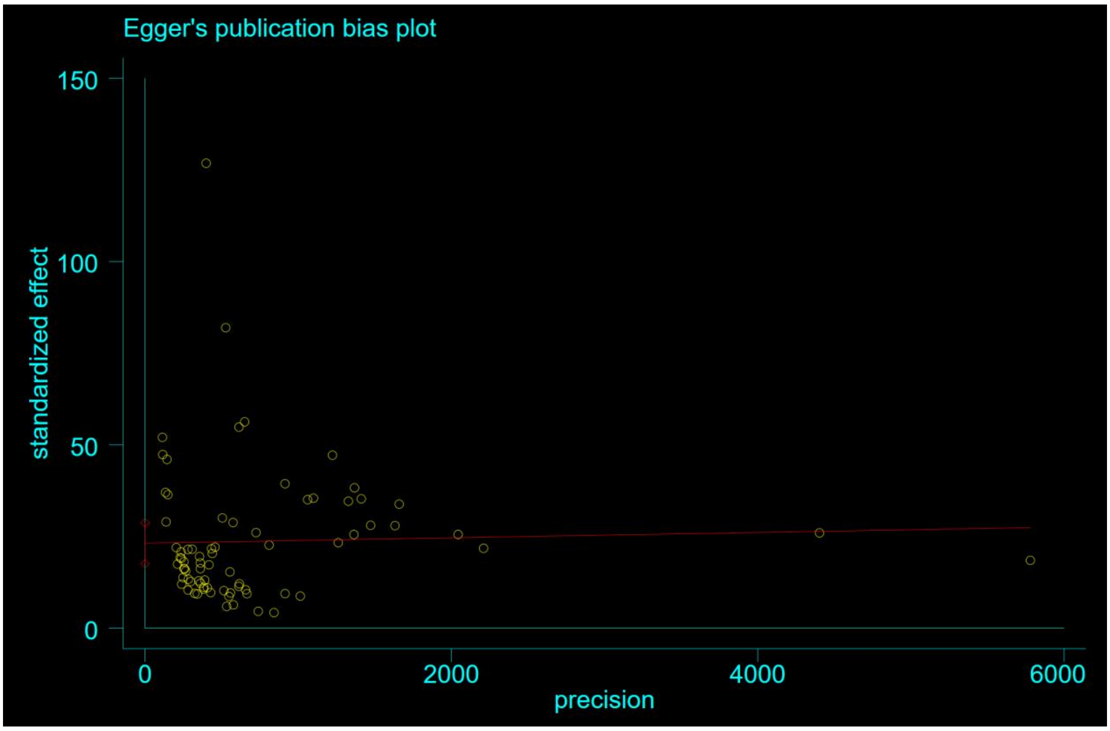

Egger's test1.

```
REML estimate of between-study variance          tau2           =  [illegible]
% residual variation due to heterogeneity        I-squared_res  =   99.85%
Proportion of between-study variance explained   Adj R-squared  =   -1.32%
With Knapp-Hartung modification
```

p	Coef.	Std. Err.	t	P>|t|	[95% Conf.	Interval]
gender_1	-.0036587	.0206352	-0.18	0.860	-.0447752	.0374577
_cons	.0672548	.0145918	4.61	0.000	.03818	.0963296

Egger's test2.

4.Region:

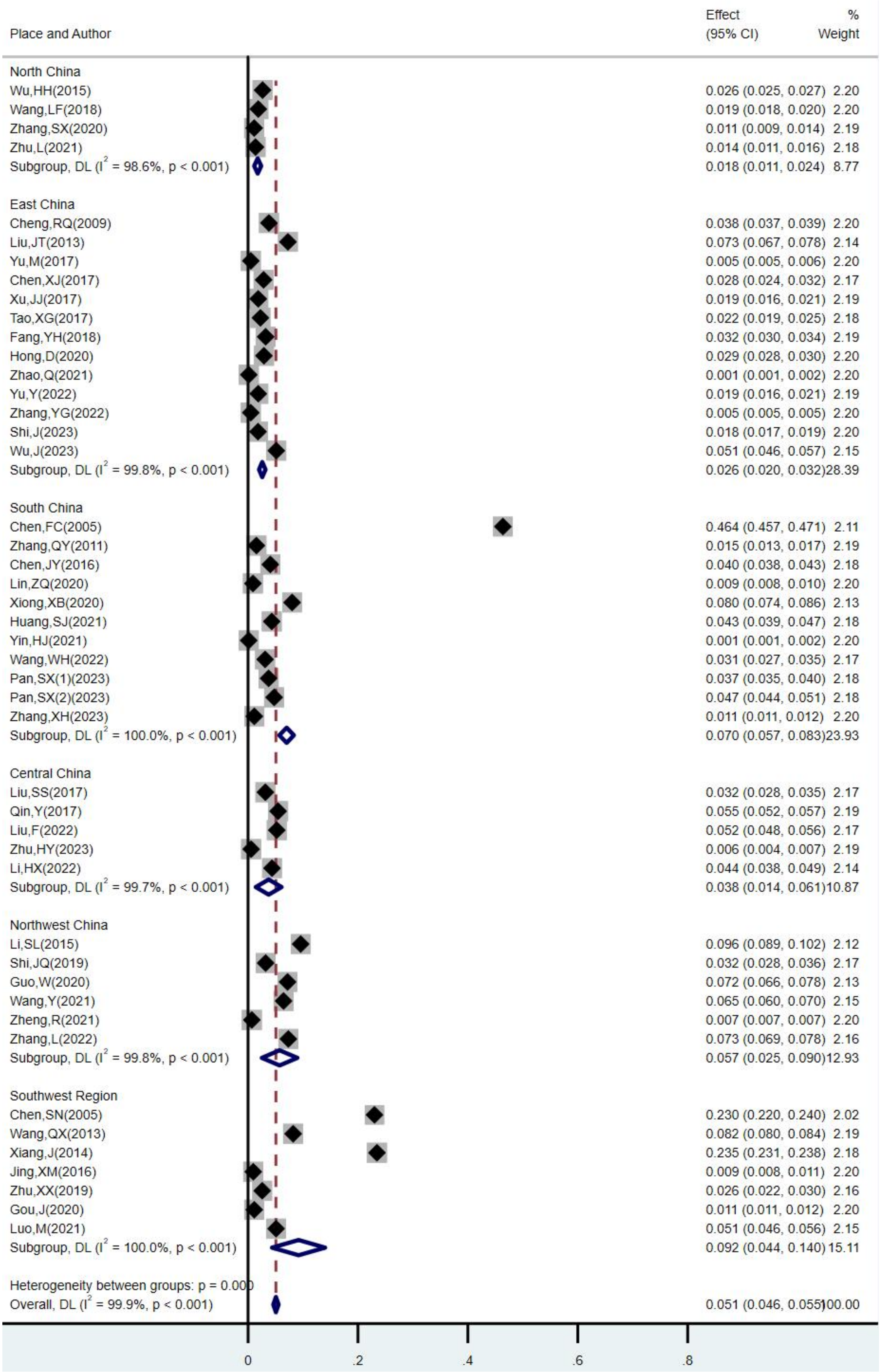

Forest Plot of meta-analysis of the Prevalence of Stunting in Region in China

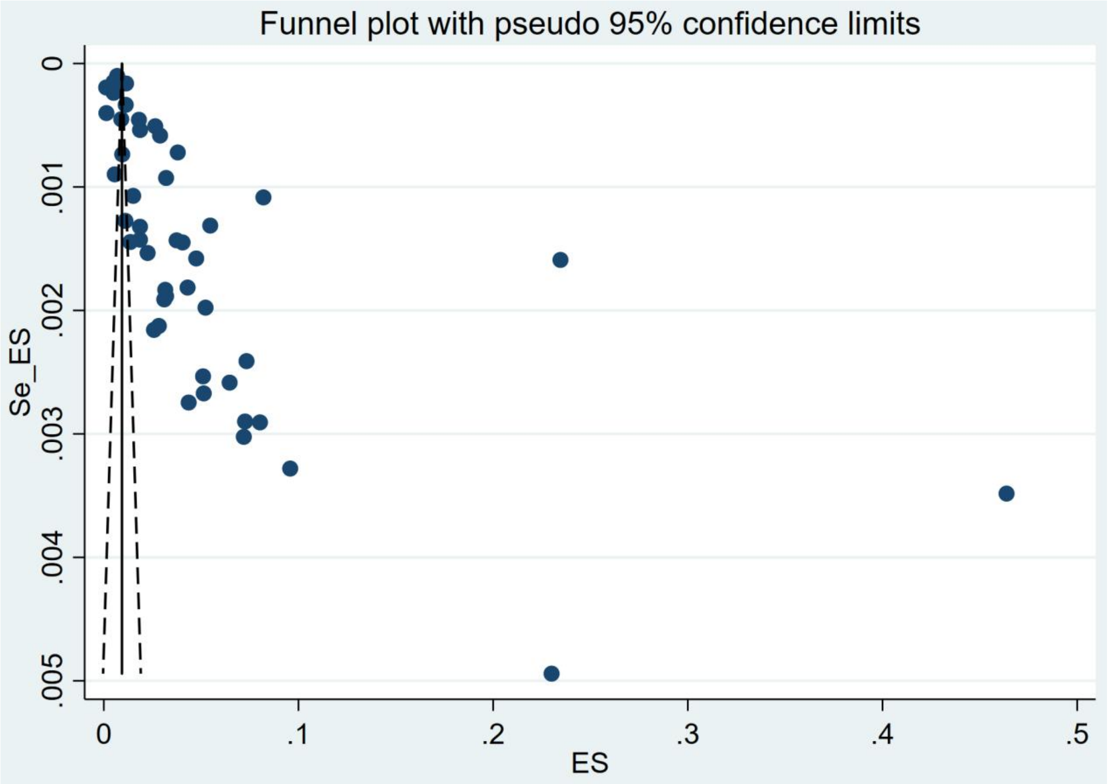

Funnel plot

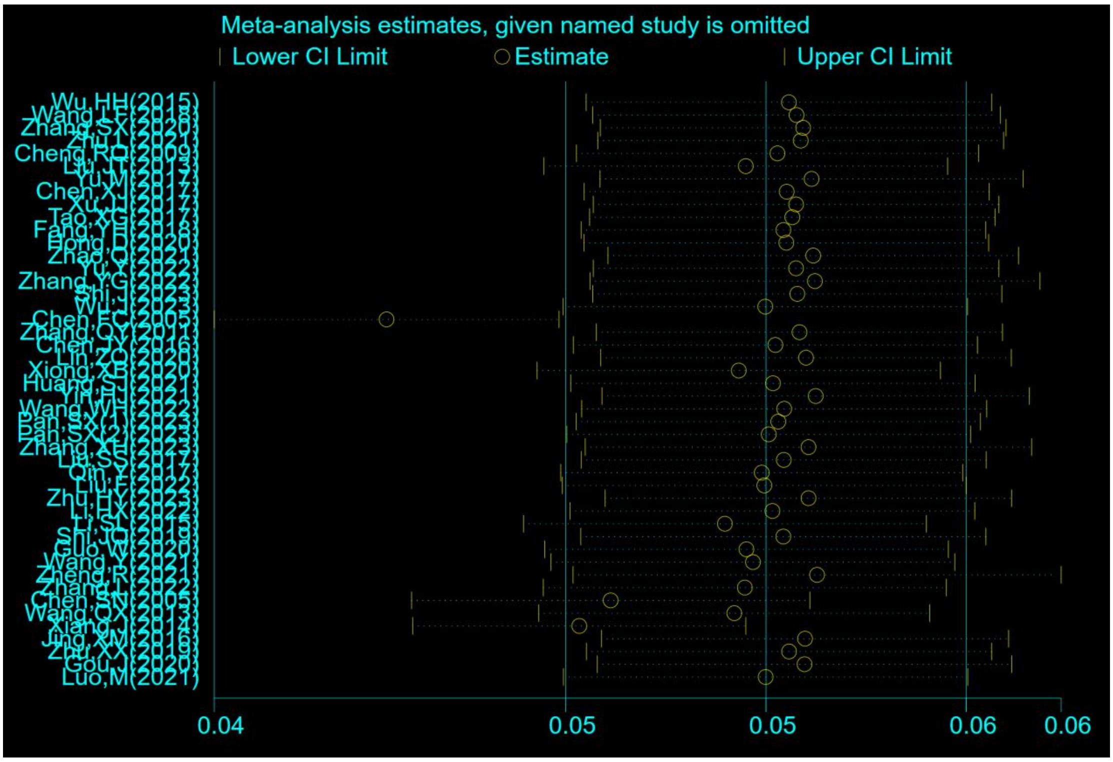

Sensitivity analysis

```
Meta-regression                                     Number of obs   =        46
REML estimate of between-study variance             tau2            =  .006113
% residual variation due to heterogeneity           I-squared_res   =   99.93%
Proportion of between-study variance explained      Adj R-squared   =   -0.33%
Joint test for all covariates                       Model F(5,40)   =     0.97
With Knapp-Hartung modification                     Prob > F        =   0.4470
```

p	Coef.	Std. Err.	t	P>|t|	[95% Conf.	Interval]
place_2	-.0113805	.0411761	-0.28	0.784	-.0946005	.0718396
place_3	-.0201053	.0524853	-0.38	0.704	-.126182	.0859714
place_4	.0198564	.0473865	0.42	0.677	-.0759154	.1156281
place_5	.0333082	.0422041	0.79	0.435	-.0519895	.1186058
place_6	.054396	.0458214	1.19	0.242	-.0382124	.1470044
_cons	.0375359	.034994	1.07	0.290	-.0331896	.1082614

Egger's test2.

5.Year:

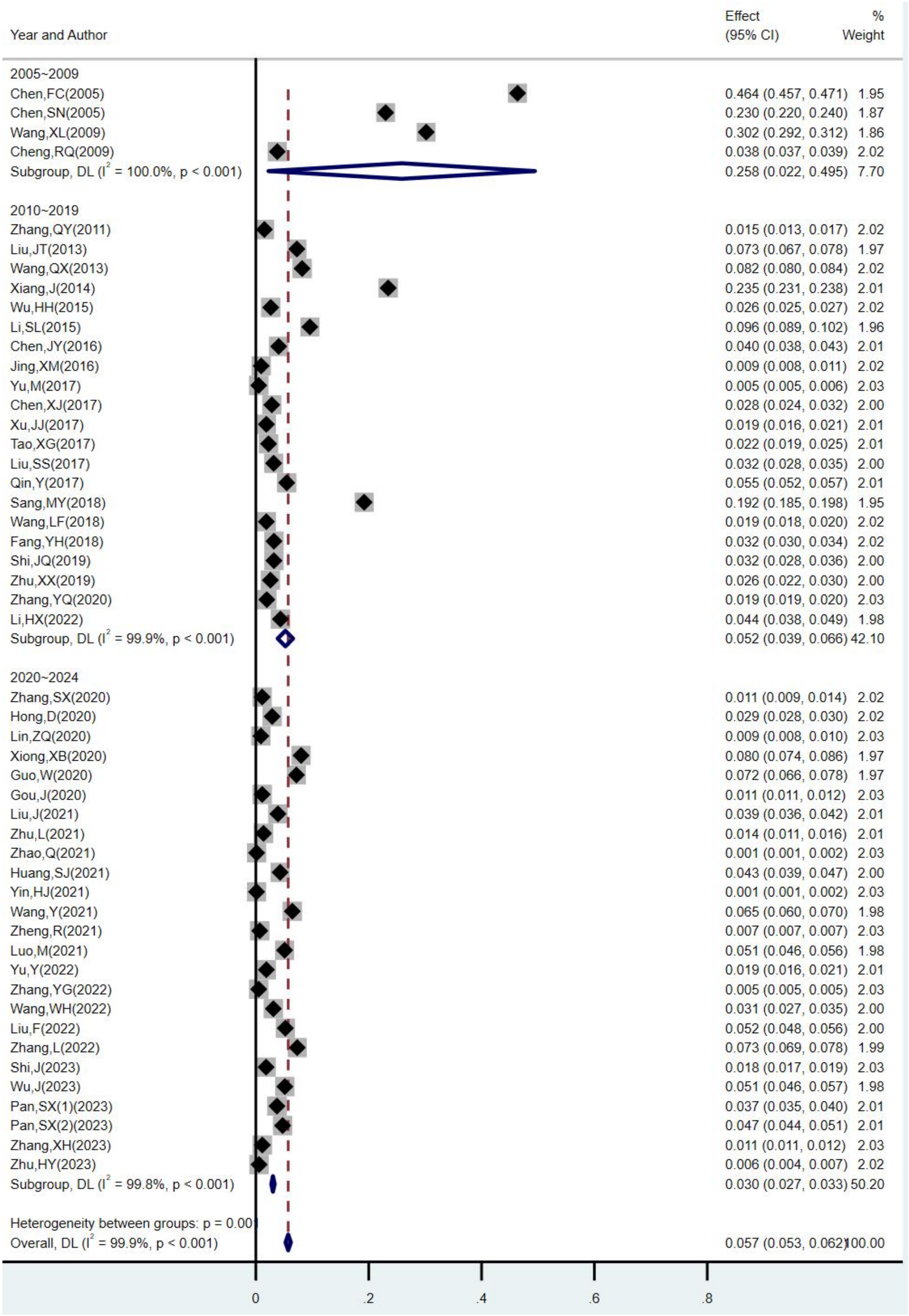

Forest Plot of meta-analysis of the Prevalence of Stunting in Year in China

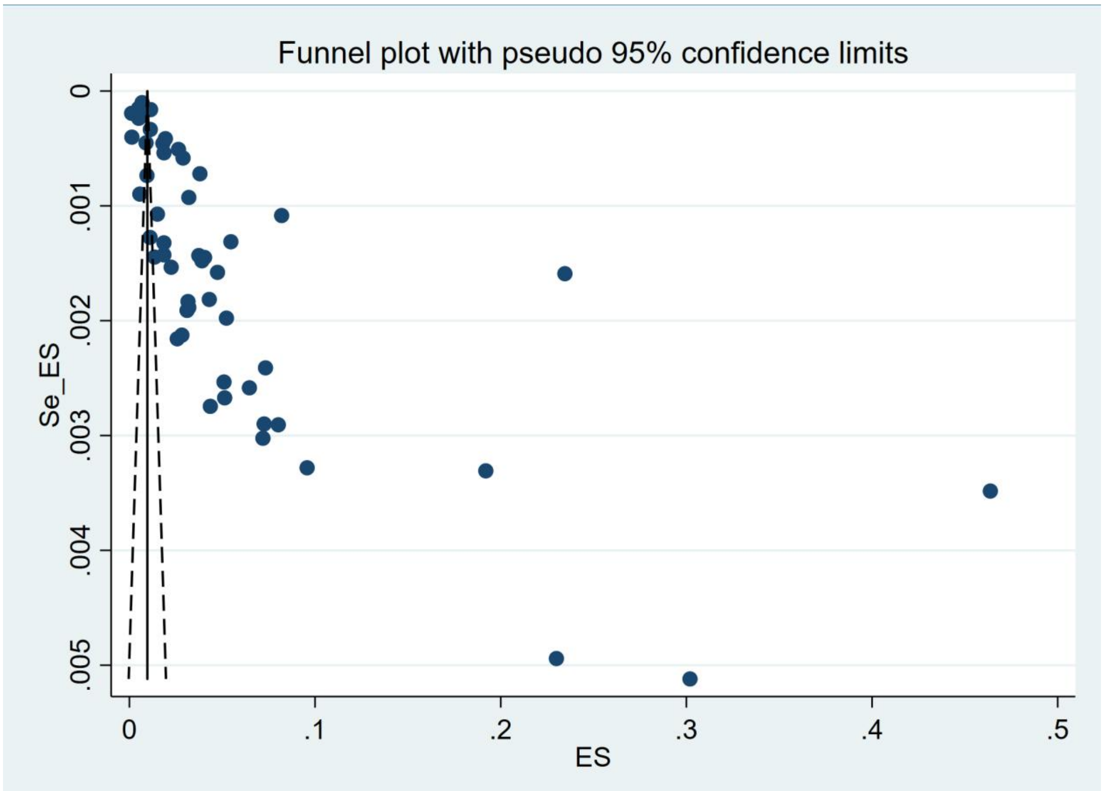

Funnel plot

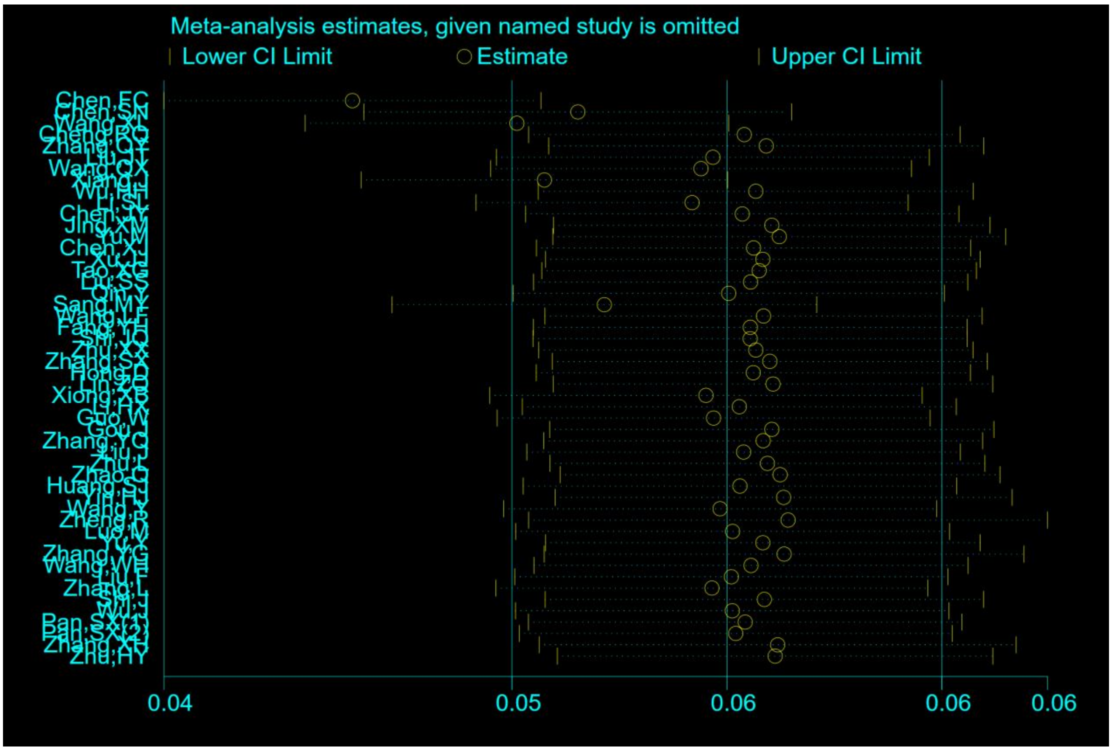

Sensitivity analysis

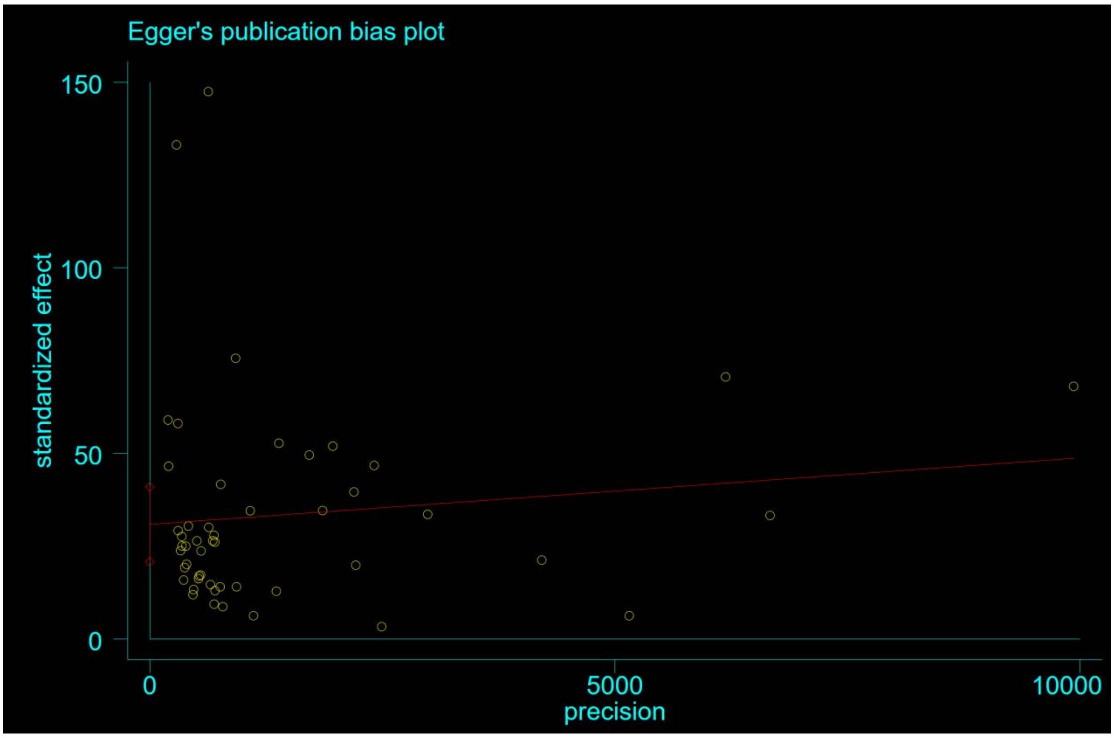

Egger's test1.

Meta-regression	Number of obs	=	50
**REML** estimate of between-study variance	tau2	=	.003743
% residual variation due to heterogeneity	I-squared_res	=	99.92%
Proportion of between-study variance explained	Adj R-squared	=	48.37%
Joint test for all covariates	Model F(2,47)	=	23.91
**With** Knapp-Hartung modification	Prob > F	=	0.0000

p	Coef.	Std. Err.	t	P>|t|	[95% Conf.	Interval]
var3_2	-.2036571	.0337253	-6.04	0.000	-.2715037	-.1358104
var3_3	-.2268564	.0328472	-6.91	0.000	-.2929364	-.1607764
_cons	.2582257	.0306617	8.42	0.000	.1965424	.3199091

Egger's test2.
