# Supplementary figures and images for: Prevalence of growth retardation among children and adolescents in China: a systematic review and meta-analysis
Source: Front Pediatr. 2025 Dec 17;13:1634605. doi: 10.3389/fped.2025.1634605 (PMC12754002; doi:10.3389/fped.2025.1634605)

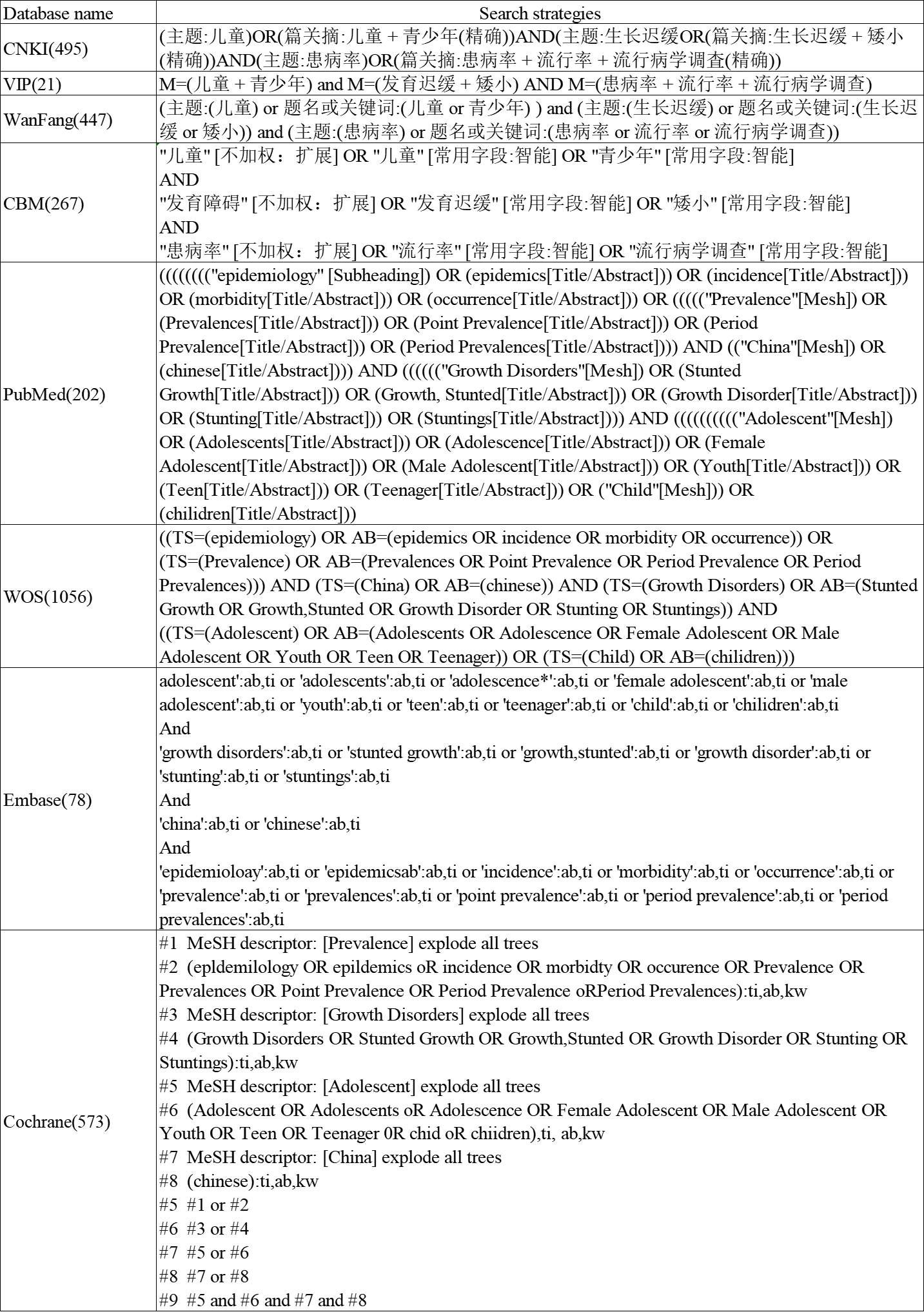

Supplement: Supplementary 2 — S2 File: Research Indicators and Their Subgroup Plots. [file Table1.docx]
